# Curcumenol from *Curcuma zedoaria*: a second monoclinic modification

**DOI:** 10.1107/S1600536810040559

**Published:** 2010-10-20

**Authors:** Omer Abdalla Ahmed Hamdi, Khalijah Awang, A. Hamid A. Hadi, Devi Rosmy Syamsir, Seik Weng Ng

**Affiliations:** a Department of Chemistry, University of Malaya, 50603 Kuala Lumpur, Malaysia

## Abstract

The title compound, systematic name 9-isopropyl­idene-2,6-dimethyl-11-oxatricyclo­[6.2.1.0^1,5^]undec-6-en-8-ol, C_15_H_22_O_2_, which crystallizes with two mol­ecules of similar conformation in the asymmetric unit, features three fused rings, two of which are five-membered and the third six-membered. Of the two five-membered rings, the one with an O atom has a distinct envelope shape (with the O atom representing the flap). The six-membered ring is also envelope-shaped as it shares a common O atom with the five-membered ring. In the crystal, the two independent mol­ecules are linked by a pair of O—H⋯O hydrogen bonds, generating a dimer.

## Related literature

For the *C*2 modification isolated from *Globba malaccensis* Ridl, see: Muangsin *et al.* (2004 ▶).
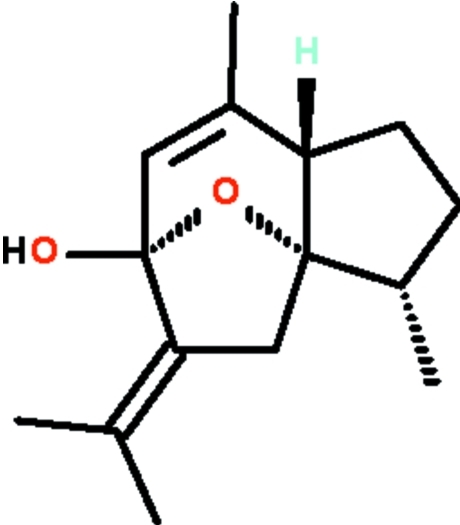


## Experimental

### 

#### Crystal data

C_15_H_22_O_2_
*M* *_r_* = 234.33
Monoclinic, 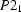

*a* = 9.3495 (7) Å
*b* = 12.535 (1) Å
*c* = 11.7727 (9) Å
β = 96.532 (1)°
*V* = 1370.76 (18) Å^3^
*Z* = 4
Mo *K*α radiation
μ = 0.07 mm^−1^
*T* = 100 K
0.40 × 0.05 × 0.05 mm

#### Data collection

Bruker SMART APEX diffractometer
13257 measured reflections
3298 independent reflections
2882 reflections with *I* > 2σ(*I*)
*R*_int_ = 0.046

#### Refinement

*R*[*F* ^2^ > 2σ(*F* ^2^)] = 0.037
*wR*(*F* ^2^) = 0.090
*S* = 1.03
3298 reflections
323 parameters
1 restraint
H atoms treated by a mixture of independent and constrained refinement
Δρ_max_ = 0.21 e Å^−3^
Δρ_min_ = −0.18 e Å^−3^

### 

Data collection: *APEX2* (Bruker, 2009 ▶); cell refinement: *SAINT* (Bruker, 2009 ▶); data reduction: *SAINT*; program(s) used to solve structure: *SHELXS97* (Sheldrick, 2008 ▶); program(s) used to refine structure: *SHELXL97* (Sheldrick, 2008 ▶); molecular graphics: *X-SEED* (Barbour, 2001 ▶); software used to prepare material for publication: *publCIF* (Westrip, 2010 ▶).

## Supplementary Material

Crystal structure: contains datablocks global, I. DOI: 10.1107/S1600536810040559/hb5676sup1.cif

Structure factors: contains datablocks I. DOI: 10.1107/S1600536810040559/hb5676Isup2.hkl

Additional supplementary materials:  crystallographic information; 3D view; checkCIF report

## Figures and Tables

**Table 1:** Hydrogen-bond geometry (Å, °)

*D*—H⋯*A*	*D*—H	H⋯*A*	*D*⋯*A*	*D*—H⋯*A*
O1—H1⋯O4	0.89 (3)	1.92 (3)	2.799 (2)	168 (3)
O3—H3⋯O2	0.86 (3)	1.92 (3)	2.771 (2)	171 (3)

**Fig. 1.**
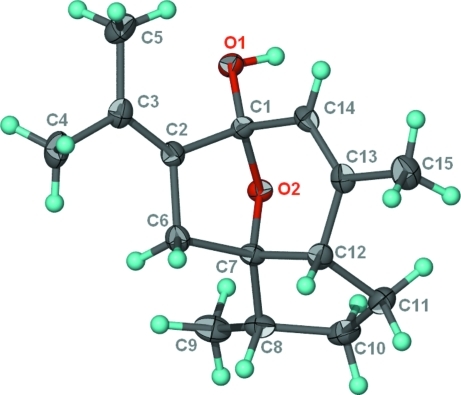
View of the first molecule of (I) at the 70% probability level; hydrogen atoms are drawn as spheres of arbitrary radius.

**Fig. 2.**
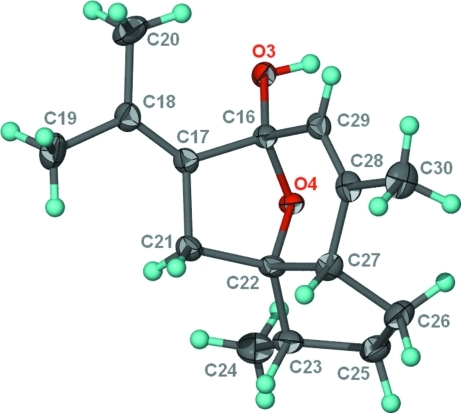
View of the second molecule of (I) at the 70% probability level; hydrogen atoms are drawn as spheres of arbitrary radius.

## supplementary crystallographic information

### Comment

*Zingerberaceae* is a herbaceous plant found in tropical forests that comprises of 52 genera with 1500 species. Most species are found in the South East Asian region. *Curcuma zedoaria*, also known as white turmeric, is a species that is a rich source of terpenoids.

Curcumenol, isolated from *Globba malaccensis* Ridl, belongs to the monoclinic, space group *C*2, with *a* 16.8467 (4), *b* 7.6799 (2), *c* 11.8613 (10) Å and *β* 115.997 (1) °. This modification is less dense, as noted from its calculated density of 1.28 (Muangsin *et al.*, 2004). The present modification (I), (Fig. 1) shows nearly identical bond dimensions in the two independent molecules. The two molecules form a dimer in the crystal, being linked by two O—H···O hydrogen bonds.

### Experimental

The rhizome of *Curcuma zedoaria* was collected from Tawangmangu, Indonesia.

Dried rhizomes (1 kg) were powdered and extracted three times with *n*-hexane and after this, with dichloromethane, ethylacetate, and methanol. The extracts were concentrated under reduced pressure given several fractions.

The *n*-hexane crude extract (20 g) was subjected to column chromatography over silica gel 60(0.063–0.200 mm, 70–230 mesh ASTM) eluted with a mixture of *n*-hexane: ethyl acetate with increasing polarity. Separation by TLC gave 21 fractions.

Fraction 10 (1.41 g) was chromatographed over silica gel (0.040–0.063 mm, mesh 230–400 ASTM) eluted with a gradient solvent system of *n*-hexane: ethyl aceate to give 5 fractions. The second fraction was further purified by high performance thin layerchromatography and using petroleum ether: ethyl acetate: acidified methanol in a 85:14:1 ratio as the developing solvent. Slow evaporation of the solvent gave (I) as colorless prisms.

### Refinement

Carbon-bound H-atoms were placed in calculated positions (C—H 0.95 to 0.98 Å) and were included in the refinement in the riding model approximation, with *U*(H) set to 1.2 to 1.5*U*(C).

In the absence of heavy atoms, 2923 Friedel pairs were merged.

The hydroxy H-atoms were located in a difference Fourier map, and were refined without restraints; their U_iso_ values were freely refined.

The absolute configuration was assumed to be that of the *C*2 modification.

### Crystal data

**Table:**

C_15_H_22_O_2_	*F*(000) = 512
*M**_r_* = 234.33	*D*_x_ = 1.135 Mg m^−^^3^
Monoclinic, *P*2_1_	Mo *K*α radiation, λ = 0.71073 Å
Hall symbol: P 2yb	Cell parameters from 3511 reflections
*a* = 9.3495 (7) Å	θ = 2.4–27.4°
*b* = 12.535 (1) Å	µ = 0.07 mm^−^^1^
*c* = 11.7727 (9) Å	*T* = 100 K
β = 96.532 (1)°	Prism, colorless
*V* = 1370.76 (18) Å^3^	0.40 × 0.05 × 0.05 mm
*Z* = 4	

### Data collection

**Table:**

Bruker SMART APEX diffractometer	2882 reflections with *I* > 2σ(*I*)
Radiation source: fine-focus sealed tube	*R*_int_ = 0.046
graphite	θ_max_ = 27.5°, θ_min_ = 2.2°
ω scans	*h* = −12→12
13257 measured reflections	*k* = −16→15
3298 independent reflections	*l* = −15→15

### Refinement

**Table:**

Refinement on *F*^2^	Primary atom site location: structure-invariant direct methods
Least-squares matrix: full	Secondary atom site location: difference Fourier map
*R*[*F*^2^ > 2σ(*F*^2^)] = 0.037	Hydrogen site location: inferred from neighbouring sites
*wR*(*F*^2^) = 0.090	H atoms treated by a mixture of independent and constrained refinement
*S* = 1.03	*w* = 1/[σ^2^(*F*_o_^2^) + (0.0535*P*)^2^ + 0.0302*P*] where *P* = (*F*_o_^2^ + 2*F*_c_^2^)/3
3298 reflections	(Δ/σ)_max_ = 0.001
323 parameters	Δρ_max_ = 0.21 e Å^−^^3^
1 restraint	Δρ_min_ = −0.18 e Å^−^^3^

### Fractional atomic coordinates and isotropic or equivalent isotropic displacement parameters (Å^2^)

**Table:**

	*x*	*y*	*z*	*U*_iso_*/*U*_eq_	
O1	0.24626 (15)	0.50024 (12)	0.49296 (13)	0.0165 (3)	
H1	0.268 (3)	0.497 (2)	0.569 (3)	0.033 (7)*	
O2	0.06839 (14)	0.62132 (11)	0.52267 (11)	0.0136 (3)	
O3	0.03989 (15)	0.49267 (12)	0.71017 (12)	0.0160 (3)	
H3	0.045 (3)	0.538 (3)	0.656 (3)	0.043 (9)*	
O4	0.28533 (14)	0.46600 (12)	0.72943 (11)	0.0137 (3)	
C1	0.2006 (2)	0.60463 (17)	0.47181 (17)	0.0141 (4)	
C2	0.1560 (2)	0.62872 (17)	0.34601 (17)	0.0153 (4)	
C3	0.2186 (2)	0.59756 (17)	0.25570 (17)	0.0178 (4)	
C4	0.1634 (3)	0.6348 (2)	0.13744 (18)	0.0266 (5)	
H4A	0.0671	0.6656	0.1381	0.040*	
H4B	0.1584	0.5741	0.0846	0.040*	
H4C	0.2287	0.6890	0.1125	0.040*	
C5	0.3508 (2)	0.52777 (19)	0.2618 (2)	0.0231 (5)	
H5A	0.3878	0.5157	0.3421	0.035*	
H5B	0.4248	0.5631	0.2226	0.035*	
H5C	0.3257	0.4592	0.2248	0.035*	
C6	0.0287 (2)	0.70366 (17)	0.34497 (17)	0.0171 (4)	
H6A	−0.0595	0.6710	0.3051	0.020*	
H6B	0.0476	0.7719	0.3070	0.020*	
C7	0.0140 (2)	0.72101 (17)	0.47172 (17)	0.0144 (4)	
C8	−0.1354 (2)	0.74213 (18)	0.50599 (18)	0.0188 (4)	
H8	−0.1786	0.8026	0.4584	0.023*	
C9	−0.2388 (2)	0.6479 (2)	0.4898 (2)	0.0252 (5)	
H9A	−0.2524	0.6276	0.4089	0.038*	
H9B	−0.3317	0.6681	0.5145	0.038*	
H9C	−0.1987	0.5874	0.5356	0.038*	
C10	−0.1057 (2)	0.7814 (2)	0.62979 (19)	0.0231 (5)	
H10A	−0.0960	0.7204	0.6834	0.028*	
H10B	−0.1851	0.8277	0.6495	0.028*	
C11	0.0359 (2)	0.84454 (19)	0.63593 (19)	0.0232 (5)	
H11A	0.1000	0.8254	0.7057	0.028*	
H11B	0.0168	0.9222	0.6371	0.028*	
C12	0.1062 (2)	0.81353 (17)	0.52712 (17)	0.0169 (4)	
H12	0.0964	0.8756	0.4734	0.020*	
C13	0.2640 (2)	0.78423 (17)	0.54836 (17)	0.0165 (4)	
C14	0.3074 (2)	0.68708 (17)	0.52234 (17)	0.0153 (4)	
H14	0.4067	0.6693	0.5357	0.018*	
C15	0.3668 (2)	0.86938 (19)	0.5957 (2)	0.0242 (5)	
H15A	0.4647	0.8402	0.6069	0.036*	
H15B	0.3395	0.8942	0.6692	0.036*	
H15C	0.3634	0.9293	0.5420	0.036*	
C16	0.1638 (2)	0.50276 (17)	0.78584 (16)	0.0139 (4)	
C17	0.1685 (2)	0.42847 (17)	0.88859 (17)	0.0151 (4)	
C18	0.0631 (2)	0.40510 (18)	0.95131 (17)	0.0185 (4)	
C19	0.0876 (2)	0.3318 (2)	1.0536 (2)	0.0269 (5)	
H19A	0.1859	0.3035	1.0596	0.040*	
H19B	0.0187	0.2726	1.0443	0.040*	
H19C	0.0740	0.3717	1.1232	0.040*	
C20	−0.0861 (2)	0.4506 (2)	0.9308 (2)	0.0283 (6)	
H20A	−0.0908	0.5024	0.8681	0.042*	
H20B	−0.1099	0.4861	1.0004	0.042*	
H20C	−0.1552	0.3930	0.9105	0.042*	
C21	0.3243 (2)	0.39168 (18)	0.91067 (17)	0.0173 (4)	
H21A	0.3322	0.3138	0.8986	0.021*	
H21B	0.3659	0.4092	0.9895	0.021*	
C22	0.3990 (2)	0.45412 (18)	0.82278 (17)	0.0154 (4)	
C23	0.5286 (2)	0.40276 (19)	0.77721 (18)	0.0199 (5)	
H23	0.5990	0.3832	0.8444	0.024*	
C24	0.4981 (3)	0.3034 (2)	0.7059 (2)	0.0280 (5)	
H24A	0.4503	0.2504	0.7499	0.042*	
H24B	0.5888	0.2738	0.6855	0.042*	
H24C	0.4354	0.3213	0.6361	0.042*	
C25	0.5938 (2)	0.4943 (2)	0.71440 (19)	0.0229 (5)	
H25A	0.5450	0.5011	0.6356	0.027*	
H25B	0.6977	0.4818	0.7105	0.027*	
C26	0.5709 (2)	0.5955 (2)	0.7840 (2)	0.0247 (5)	
H26A	0.5388	0.6555	0.7327	0.030*	
H26B	0.6613	0.6162	0.8310	0.030*	
C27	0.4527 (2)	0.56609 (18)	0.86173 (17)	0.0172 (4)	
H27	0.4996	0.5604	0.9422	0.021*	
C28	0.3310 (2)	0.64559 (18)	0.85997 (17)	0.0176 (4)	
C29	0.1969 (2)	0.61599 (17)	0.82481 (17)	0.0160 (4)	
H29	0.1210	0.6664	0.8241	0.019*	
C30	0.3704 (3)	0.75649 (19)	0.9014 (2)	0.0253 (5)	
H30A	0.2833	0.8004	0.8977	0.038*	
H30B	0.4378	0.7880	0.8529	0.038*	
H30C	0.4158	0.7532	0.9806	0.038*	

### Atomic displacement parameters (Å^2^)

**Table:**

	*U*^11^	*U*^22^	*U*^33^	*U*^12^	*U*^13^	*U*^23^
O1	0.0223 (7)	0.0128 (7)	0.0142 (8)	0.0047 (6)	0.0011 (6)	0.0017 (6)
O2	0.0151 (6)	0.0123 (7)	0.0137 (7)	0.0011 (5)	0.0026 (5)	0.0029 (6)
O3	0.0140 (7)	0.0201 (8)	0.0134 (7)	−0.0019 (6)	−0.0002 (5)	0.0034 (6)
O4	0.0115 (6)	0.0172 (8)	0.0123 (7)	0.0015 (6)	0.0014 (5)	0.0005 (6)
C1	0.0155 (9)	0.0134 (10)	0.0136 (9)	0.0016 (8)	0.0027 (7)	0.0000 (8)
C2	0.0168 (9)	0.0130 (10)	0.0158 (10)	−0.0015 (8)	0.0002 (7)	0.0001 (8)
C3	0.0241 (10)	0.0146 (10)	0.0150 (10)	−0.0019 (9)	0.0029 (8)	0.0000 (8)
C4	0.0398 (13)	0.0256 (12)	0.0142 (10)	0.0004 (11)	0.0028 (9)	−0.0020 (9)
C5	0.0294 (12)	0.0206 (12)	0.0209 (11)	0.0032 (9)	0.0097 (9)	0.0001 (9)
C6	0.0192 (10)	0.0173 (11)	0.0144 (10)	−0.0004 (8)	0.0003 (8)	0.0017 (8)
C7	0.0150 (9)	0.0121 (10)	0.0157 (10)	0.0020 (8)	0.0006 (7)	0.0032 (8)
C8	0.0176 (10)	0.0192 (12)	0.0200 (10)	0.0042 (8)	0.0045 (8)	0.0044 (9)
C9	0.0153 (10)	0.0272 (13)	0.0333 (13)	0.0004 (9)	0.0043 (9)	0.0051 (10)
C10	0.0243 (11)	0.0230 (12)	0.0231 (11)	0.0077 (9)	0.0080 (9)	0.0025 (9)
C11	0.0266 (11)	0.0226 (13)	0.0201 (11)	0.0070 (10)	0.0012 (9)	−0.0038 (9)
C12	0.0229 (10)	0.0127 (10)	0.0147 (10)	0.0015 (8)	0.0012 (8)	0.0020 (8)
C13	0.0203 (10)	0.0153 (11)	0.0138 (10)	−0.0030 (8)	0.0011 (8)	0.0028 (8)
C14	0.0139 (9)	0.0190 (11)	0.0128 (10)	−0.0013 (8)	0.0011 (7)	0.0036 (8)
C15	0.0260 (11)	0.0192 (12)	0.0263 (12)	−0.0064 (9)	−0.0014 (9)	0.0006 (10)
C16	0.0130 (9)	0.0163 (10)	0.0122 (9)	0.0007 (8)	0.0016 (7)	0.0002 (8)
C17	0.0159 (9)	0.0152 (10)	0.0134 (9)	−0.0011 (8)	−0.0015 (8)	0.0002 (8)
C18	0.0192 (10)	0.0214 (12)	0.0144 (10)	−0.0023 (9)	−0.0003 (8)	0.0024 (9)
C19	0.0257 (12)	0.0342 (14)	0.0205 (11)	−0.0088 (10)	0.0016 (9)	0.0083 (10)
C20	0.0165 (11)	0.0448 (16)	0.0241 (12)	−0.0011 (11)	0.0049 (9)	0.0047 (11)
C21	0.0172 (10)	0.0199 (11)	0.0144 (10)	0.0026 (9)	0.0010 (8)	0.0033 (8)
C22	0.0123 (9)	0.0213 (11)	0.0119 (10)	0.0018 (8)	−0.0023 (7)	0.0017 (8)
C23	0.0155 (10)	0.0271 (13)	0.0173 (10)	0.0053 (9)	0.0021 (8)	0.0033 (9)
C24	0.0269 (11)	0.0262 (14)	0.0322 (13)	0.0075 (10)	0.0096 (10)	−0.0035 (10)
C25	0.0163 (10)	0.0322 (13)	0.0206 (11)	0.0011 (9)	0.0040 (8)	0.0044 (10)
C26	0.0169 (10)	0.0300 (13)	0.0272 (12)	−0.0034 (9)	0.0034 (9)	0.0039 (10)
C27	0.0158 (10)	0.0238 (12)	0.0114 (10)	−0.0031 (8)	−0.0008 (8)	−0.0002 (8)
C28	0.0228 (10)	0.0179 (11)	0.0128 (10)	−0.0004 (9)	0.0041 (8)	−0.0005 (8)
C29	0.0183 (9)	0.0151 (11)	0.0150 (10)	0.0018 (8)	0.0044 (8)	0.0014 (8)
C30	0.0295 (12)	0.0221 (12)	0.0240 (12)	−0.0065 (10)	0.0023 (9)	−0.0060 (10)

### Geometric parameters (Å, °)

**Table:**

O1—C1	1.390 (3)	C14—H14	0.9500
O1—H1	0.89 (3)	C15—H15A	0.9800
O2—C1	1.449 (2)	C15—H15B	0.9800
O2—C7	1.453 (2)	C15—H15C	0.9800
O3—C16	1.385 (2)	C16—C29	1.513 (3)
O3—H3	0.86 (3)	C16—C17	1.523 (3)
O4—C22	1.447 (2)	C17—C18	1.329 (3)
O4—C16	1.455 (2)	C17—C21	1.522 (3)
C1—C14	1.511 (3)	C18—C20	1.501 (3)
C1—C2	1.523 (3)	C18—C19	1.512 (3)
C2—C3	1.329 (3)	C19—H19A	0.9800
C2—C6	1.515 (3)	C19—H19B	0.9800
C3—C4	1.503 (3)	C19—H19C	0.9800
C3—C5	1.510 (3)	C20—H20A	0.9800
C4—H4A	0.9800	C20—H20B	0.9800
C4—H4B	0.9800	C20—H20C	0.9800
C4—H4C	0.9800	C21—C22	1.529 (3)
C5—H5A	0.9800	C21—H21A	0.9900
C5—H5B	0.9800	C21—H21B	0.9900
C5—H5C	0.9800	C22—C23	1.522 (3)
C6—C7	1.530 (3)	C22—C27	1.542 (3)
C6—H6A	0.9900	C23—C24	1.511 (3)
C6—H6B	0.9900	C23—C25	1.529 (3)
C7—C8	1.521 (3)	C23—H23	1.0000
C7—C12	1.545 (3)	C24—H24A	0.9800
C8—C9	1.524 (3)	C24—H24B	0.9800
C8—C10	1.534 (3)	C24—H24C	0.9800
C8—H8	1.0000	C25—C26	1.539 (3)
C9—H9A	0.9800	C25—H25A	0.9900
C9—H9B	0.9800	C25—H25B	0.9900
C9—H9C	0.9800	C26—C27	1.557 (3)
C10—C11	1.537 (3)	C26—H26A	0.9900
C10—H10A	0.9900	C26—H26B	0.9900
C10—H10B	0.9900	C27—C28	1.511 (3)
C11—C12	1.554 (3)	C27—H27	1.0000
C11—H11A	0.9900	C28—C29	1.328 (3)
C11—H11B	0.9900	C28—C30	1.505 (3)
C12—C13	1.514 (3)	C29—H29	0.9500
C12—H12	1.0000	C30—H30A	0.9800
C13—C14	1.330 (3)	C30—H30B	0.9800
C13—C15	1.500 (3)	C30—H30C	0.9800
			
C1—O1—H1	105 (2)	H15A—C15—H15C	109.5
C1—O2—C7	103.23 (13)	H15B—C15—H15C	109.5
C16—O3—H3	108 (2)	O3—C16—O4	108.49 (15)
C22—O4—C16	103.31 (13)	O3—C16—C29	114.09 (17)
O1—C1—O2	108.73 (15)	O4—C16—C29	106.99 (15)
O1—C1—C14	113.42 (17)	O3—C16—C17	113.64 (17)
O2—C1—C14	107.18 (16)	O4—C16—C17	102.46 (15)
O1—C1—C2	113.92 (17)	C29—C16—C17	110.27 (16)
O2—C1—C2	102.77 (15)	C18—C17—C21	126.42 (19)
C14—C1—C2	110.05 (16)	C18—C17—C16	128.31 (19)
C3—C2—C6	126.34 (18)	C21—C17—C16	105.13 (16)
C3—C2—C1	128.53 (19)	C17—C18—C20	124.1 (2)
C6—C2—C1	105.00 (16)	C17—C18—C19	121.5 (2)
C2—C3—C4	120.92 (19)	C20—C18—C19	114.39 (18)
C2—C3—C5	124.38 (19)	C18—C19—H19A	109.5
C4—C3—C5	114.66 (17)	C18—C19—H19B	109.5
C3—C4—H4A	109.5	H19A—C19—H19B	109.5
C3—C4—H4B	109.5	C18—C19—H19C	109.5
H4A—C4—H4B	109.5	H19A—C19—H19C	109.5
C3—C4—H4C	109.5	H19B—C19—H19C	109.5
H4A—C4—H4C	109.5	C18—C20—H20A	109.5
H4B—C4—H4C	109.5	C18—C20—H20B	109.5
C3—C5—H5A	109.5	H20A—C20—H20B	109.5
C3—C5—H5B	109.5	C18—C20—H20C	109.5
H5A—C5—H5B	109.5	H20A—C20—H20C	109.5
C3—C5—H5C	109.5	H20B—C20—H20C	109.5
H5A—C5—H5C	109.5	C17—C21—C22	103.41 (16)
H5B—C5—H5C	109.5	C17—C21—H21A	111.1
C2—C6—C7	103.77 (16)	C22—C21—H21A	111.1
C2—C6—H6A	111.0	C17—C21—H21B	111.1
C7—C6—H6A	111.0	C22—C21—H21B	111.1
C2—C6—H6B	111.0	H21A—C21—H21B	109.0
C7—C6—H6B	111.0	O4—C22—C23	108.73 (16)
H6A—C6—H6B	109.0	O4—C22—C21	102.34 (15)
O2—C7—C8	109.23 (15)	C23—C22—C21	117.75 (18)
O2—C7—C6	102.38 (16)	O4—C22—C27	108.54 (16)
C8—C7—C6	118.09 (17)	C23—C22—C27	104.17 (16)
O2—C7—C12	108.61 (15)	C21—C22—C27	115.02 (16)
C8—C7—C12	104.05 (16)	C24—C23—C22	115.91 (18)
C6—C7—C12	114.25 (16)	C24—C23—C25	114.24 (18)
C7—C8—C9	114.86 (18)	C22—C23—C25	103.31 (18)
C7—C8—C10	103.63 (17)	C24—C23—H23	107.7
C9—C8—C10	114.29 (18)	C22—C23—H23	107.7
C7—C8—H8	107.9	C25—C23—H23	107.7
C9—C8—H8	107.9	C23—C24—H24A	109.5
C10—C8—H8	107.9	C23—C24—H24B	109.5
C8—C9—H9A	109.5	H24A—C24—H24B	109.5
C8—C9—H9B	109.5	C23—C24—H24C	109.5
H9A—C9—H9B	109.5	H24A—C24—H24C	109.5
C8—C9—H9C	109.5	H24B—C24—H24C	109.5
H9A—C9—H9C	109.5	C23—C25—C26	105.89 (17)
H9B—C9—H9C	109.5	C23—C25—H25A	110.6
C8—C10—C11	105.77 (17)	C26—C25—H25A	110.6
C8—C10—H10A	110.6	C23—C25—H25B	110.6
C11—C10—H10A	110.6	C26—C25—H25B	110.6
C8—C10—H10B	110.6	H25A—C25—H25B	108.7
C11—C10—H10B	110.6	C25—C26—C27	105.59 (19)
H10A—C10—H10B	108.7	C25—C26—H26A	110.6
C10—C11—C12	105.99 (18)	C27—C26—H26A	110.6
C10—C11—H11A	110.5	C25—C26—H26B	110.6
C12—C11—H11A	110.5	C27—C26—H26B	110.6
C10—C11—H11B	110.5	H26A—C26—H26B	108.8
C12—C11—H11B	110.5	C28—C27—C22	112.18 (17)
H11A—C11—H11B	108.7	C28—C27—C26	114.80 (18)
C13—C12—C7	111.99 (17)	C22—C27—C26	105.62 (17)
C13—C12—C11	114.85 (17)	C28—C27—H27	108.0
C7—C12—C11	105.52 (17)	C22—C27—H27	108.0
C13—C12—H12	108.1	C26—C27—H27	108.0
C7—C12—H12	108.1	C29—C28—C30	123.2 (2)
C11—C12—H12	108.1	C29—C28—C27	120.2 (2)
C14—C13—C15	122.5 (2)	C30—C28—C27	116.66 (18)
C14—C13—C12	120.07 (19)	C28—C29—C16	120.68 (18)
C15—C13—C12	117.38 (19)	C28—C29—H29	119.7
C13—C14—C1	120.76 (19)	C16—C29—H29	119.7
C13—C14—H14	119.6	C28—C30—H30A	109.5
C1—C14—H14	119.6	C28—C30—H30B	109.5
C13—C15—H15A	109.5	H30A—C30—H30B	109.5
C13—C15—H15B	109.5	C28—C30—H30C	109.5
H15A—C15—H15B	109.5	H30A—C30—H30C	109.5
C13—C15—H15C	109.5	H30B—C30—H30C	109.5
			
C7—O2—C1—O1	−166.41 (15)	C22—O4—C16—O3	−165.83 (16)
C7—O2—C1—C14	70.62 (18)	C22—O4—C16—C29	70.65 (18)
C7—O2—C1—C2	−45.36 (18)	C22—O4—C16—C17	−45.36 (18)
O1—C1—C2—C3	−41.0 (3)	O3—C16—C17—C18	−42.5 (3)
O2—C1—C2—C3	−158.4 (2)	O4—C16—C17—C18	−159.3 (2)
C14—C1—C2—C3	87.7 (3)	C29—C16—C17—C18	87.1 (3)
O1—C1—C2—C6	142.99 (17)	O3—C16—C17—C21	141.52 (17)
O2—C1—C2—C6	25.6 (2)	O4—C16—C17—C21	24.7 (2)
C14—C1—C2—C6	−88.34 (19)	C29—C16—C17—C21	−88.93 (19)
C6—C2—C3—C4	−1.1 (3)	C21—C17—C18—C20	175.0 (2)
C1—C2—C3—C4	−176.4 (2)	C16—C17—C18—C20	−0.2 (4)
C6—C2—C3—C5	176.2 (2)	C21—C17—C18—C19	−2.8 (4)
C1—C2—C3—C5	1.0 (4)	C16—C17—C18—C19	−178.0 (2)
C3—C2—C6—C7	−173.7 (2)	C18—C17—C21—C22	−172.6 (2)
C1—C2—C6—C7	2.5 (2)	C16—C17—C21—C22	3.6 (2)
C1—O2—C7—C8	172.96 (16)	C16—O4—C22—C23	173.18 (17)
C1—O2—C7—C6	47.00 (17)	C16—O4—C22—C21	47.91 (18)
C1—O2—C7—C12	−74.17 (17)	C16—O4—C22—C27	−74.09 (17)
C2—C6—C7—O2	−29.52 (19)	C17—C21—C22—O4	−30.8 (2)
C2—C6—C7—C8	−149.48 (18)	C17—C21—C22—C23	−149.86 (18)
C2—C6—C7—C12	87.7 (2)	C17—C21—C22—C27	86.7 (2)
O2—C7—C8—C9	−48.6 (2)	O4—C22—C23—C24	−49.5 (2)
C6—C7—C8—C9	67.7 (2)	C21—C22—C23—C24	66.2 (3)
C12—C7—C8—C9	−164.47 (18)	C27—C22—C23—C24	−165.10 (18)
O2—C7—C8—C10	76.7 (2)	O4—C22—C23—C25	76.2 (2)
C6—C7—C8—C10	−166.94 (19)	C21—C22—C23—C25	−168.10 (18)
C12—C7—C8—C10	−39.1 (2)	C27—C22—C23—C25	−39.4 (2)
C7—C8—C10—C11	34.1 (2)	C24—C23—C25—C26	162.08 (18)
C9—C8—C10—C11	159.84 (18)	C22—C23—C25—C26	35.3 (2)
C8—C10—C11—C12	−15.7 (2)	C23—C25—C26—C27	−17.4 (2)
O2—C7—C12—C13	38.7 (2)	O4—C22—C27—C28	38.7 (2)
C8—C7—C12—C13	155.00 (16)	C23—C22—C27—C28	154.41 (17)
C6—C7—C12—C13	−74.8 (2)	C21—C22—C27—C28	−75.2 (2)
O2—C7—C12—C11	−86.90 (18)	O4—C22—C27—C26	−87.07 (18)
C8—C7—C12—C11	29.4 (2)	C23—C22—C27—C26	28.7 (2)
C6—C7—C12—C11	159.54 (17)	C21—C22—C27—C26	159.03 (17)
C10—C11—C12—C13	−132.17 (19)	C25—C26—C27—C28	−131.0 (2)
C10—C11—C12—C7	−8.3 (2)	C25—C26—C27—C22	−6.9 (2)
C7—C12—C13—C14	−1.7 (3)	C22—C27—C28—C29	−1.6 (3)
C11—C12—C13—C14	118.7 (2)	C26—C27—C28—C29	119.0 (2)
C7—C12—C13—C15	176.55 (17)	C22—C27—C28—C30	177.41 (17)
C11—C12—C13—C15	−63.1 (2)	C26—C27—C28—C30	−62.0 (2)
C15—C13—C14—C1	−178.33 (18)	C30—C28—C29—C16	−179.05 (19)
C12—C13—C14—C1	−0.2 (3)	C27—C28—C29—C16	−0.1 (3)
O1—C1—C14—C13	−154.67 (18)	O3—C16—C29—C28	−154.60 (18)
O2—C1—C14—C13	−34.7 (2)	O4—C16—C29—C28	−34.6 (2)
C2—C1—C14—C13	76.4 (2)	C17—C16—C29—C28	76.1 (2)

### Hydrogen-bond geometry (Å, °)

**Table:**

*D*—H···*A*	*D*—H	H···*A*	*D*···*A*	*D*—H···*A*
O1—H1···O4	0.89 (3)	1.92 (3)	2.799 (2)	168 (3)
O3—H3···O2	0.86 (3)	1.92 (3)	2.771 (2)	171 (3)
